# Synthesis and characterization of new hydrolytic-resistant dental resin adhesive monomer HMTAF

**DOI:** 10.1080/15685551.2019.1615789

**Published:** 2019-05-12

**Authors:** Nattawut Decha, Supitcha Talungchit, Panata Iawsipo, Arthit Pikulngam, Piangkwan Saiprasert, Chittreeya Tansakul

**Affiliations:** a Department of Chemistry and Center of Excellence for Innovation in Chemistry, Faculty of Science, Prince of Songkla University, Hat Yai, Thailand; b Department of Conservative Dentistry, Faculty of Dentistry, Prince of Songkla University, Hat Yai, Thailand; c Department of Biochemistry and Center of Excellence for Innovation in Chemistry, Faculty of Science, Burapha University, Chonburi, Thailand

**Keywords:** Methacrylamide, quaternary ammonium salt, resin adhesive, hydrolytic resistance, antibacterial monomer

## Abstract

Hydrolytic and enzymatic degradation of resin adhesives over time has been mainly attributed to secondary caries formation of methacrylate-based tooth-colored resin-based composite restorations. Ability of resin adhesive monomers to infiltrate into demineralized dentin forming stiff polymer matrix and potentially bonding to tooth structure is also a crucial property. The only commercially available antibacterial monomer, 12-methacryloyloxydodecyl pyridinium bromide (MDPB), is a quaternary ammonium methacrylate. This methacrylate monomer undergoes hydrolytic degradation, and could not bond to tooth structure. In this study, a new hydrolytic resistant monomer **HMTAF** was synthesized. It is methacrylamide-based monomer that, unlike methacrylate, is highly resistant to hydrolysis. Its molecular structure has particular functional groups; quaternary ammonium fluoride salt with potential antibacterial fluoride-releasing activity, hydroxyl and amide group with hydrogen bonding potential to dentin collagen. Hydroxyl group also increases monomer hydrophilicity for better penetration into water-saturated dentin and sufficient resin-dentin bond. The synthesized **HMTAF** and its polymer showed no hydrolytic degradation in acidic environment, while MDPB and its polymer were partially decomposed under this challenge. The conversion of monomer **HMTAF** to polymer was illustrated by FT-IR. The results indicated that **HMTAF** is highly resistant to hydrolysis, polymerizable and non-cytotoxic to Vero cell lines. It is a potential monomer to be incorporated into resin adhesives for improving hydrolytic and enzymatic resistance.

## Introduction

**Figure 1.**
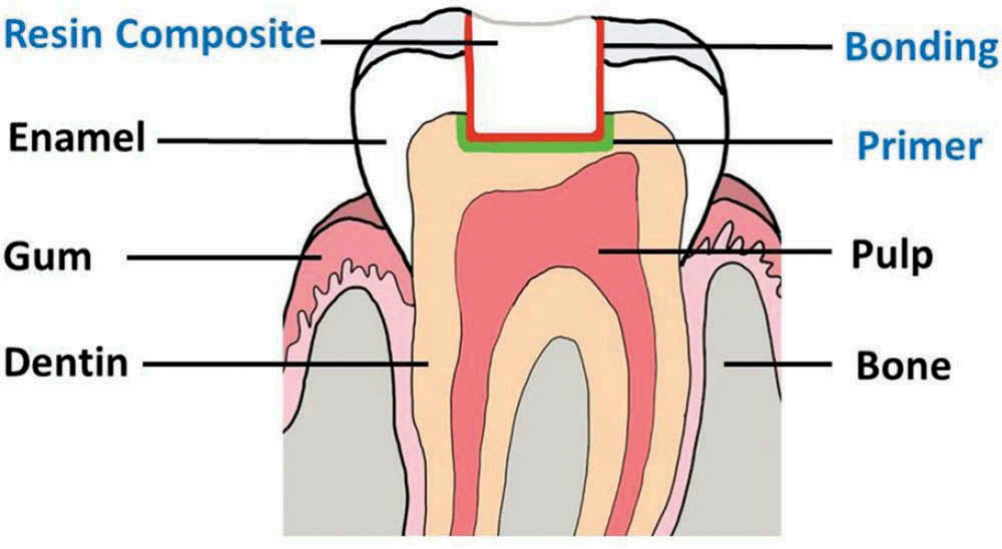
Illustration of the tooth and position of a primer, bonding, and resin composite.

Tooth-colored resin-based composite has been increasingly used in clinical practice due to esthetic concerns and the phase down of amalgam because of its toxicity. However, this material becomes defective and requires replacement nearly twice as often as amalgam [1,2]. Moreover, the cost for placing resin-based composite is about 1.5 times that of placing amalgam. Since the phase down of amalgam could not be refused, improving the longevity of resin-based composite restorations is essential. Three-step etch-and-rinse adhesive system remains the gold standard for bonding resin-based composite restorative materials to tooth structure [3]. The three steps consist of the application of an etchant, primer, and bonding agent, respectively (Figure 1). While bonding to enamel is effective, bonding to dentin is compromised due to collagen and moisture rich nature of dentin. After etching with 35–37% phosphoric acid and water rinsing, inorganic part of dentin is demineralized leaving water-saturated collagen matrix for subsequently bonding. A primer is then applied into the collagen matrix followed by light air-blow. Solvents in primers accelerate water evaporation from the collagen matrix during the air-blow, while monomers in primers penetrate in-between collagen fibrils. Last, a thin layer of bonding is applied to cover all cavity walls and light cured using visible blue light. Upon light curing, monomers in a bonding are co-polymerized with monomers in a primer providing micromechanical lock to the tooth structure before placing a resin composite [4]. Ideally, monomers in primers should be relatively hydrophilic for efficient infiltration into water-saturated dentin and able to bond to hydroxyapatite or collagen in dentin. Monomers in a bonding should be relatively hydrophobic and have cross-linking potential for strong and durable bond to tooth structure.

**Scheme 1.**
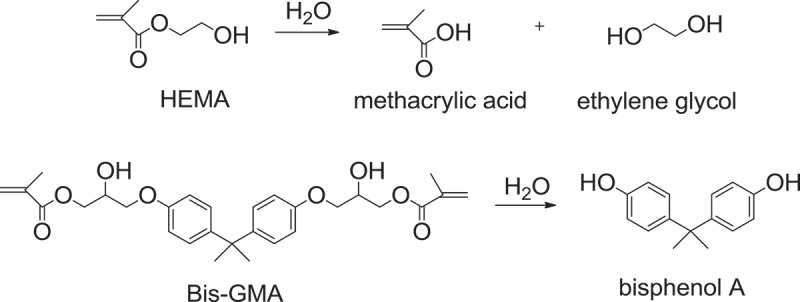
Hydrolysis reactions of HEMA and Bis-GMA.

Previous studies reported that the failure of resin-based composite restoration was mainly due to the marginal leakage at the resin-tooth interface resulting in secondary caries formation [5,6]. The average lifetime of amalgam is about 8.7 years, while the lifetime of resin adhesive is only about 5 years [2]. Hydrolytic and enzymatic degradation of ester bond of resin adhesives and lacking of their antibacterial property are main causes of short lifetime of resin-based composite [7]. Esterase is a hydrolase enzyme produced by cariogenic bacteria that breaks down the ester bonds in methacrylate monomers. As a consequence, hydrolytic resistance, antibacterial property, and ability of monomers to infiltrate into collagen matrix and form stiff and durable resin matrix are required in order to extend longevity of resin-based composite restorations. To prevent hydrolytic degradation, relatively hydrophobic monomers, such as Bis-GMA and TEGDMA, are preferred over hydrophilic monomers, such as HEMA. However, hydrophobic monomers are not efficiently infiltrated into hydrophilic demineralized collagen matrix. Consequently, hydrophilic monomers are still incorporated in the primer in order to facilitate monomer infiltration[8]. Commonly used resin adhesives are acrylates, usually methacrylates, which can be hydrolyzed in aqueous environment of the oral cavity [9]. For example, hydrolysis of HEMA provides methacrylic acid and ethylene glycol, and combined hydrolytic and enzymatic degradation of Bis-GMA affords bisphenol A which is harmful for the reproductive system (Scheme 1) [10]. There was a report that unpolymerized HEMA may cause allergy [11]. Nevertheless, Bis-GMA and HEMA are still popularly used. Bis-GMA forms stiff resin matrix and HEMA is biocompatible and greatly soluble in ethanol and acetone [12].

**Figure 2.**
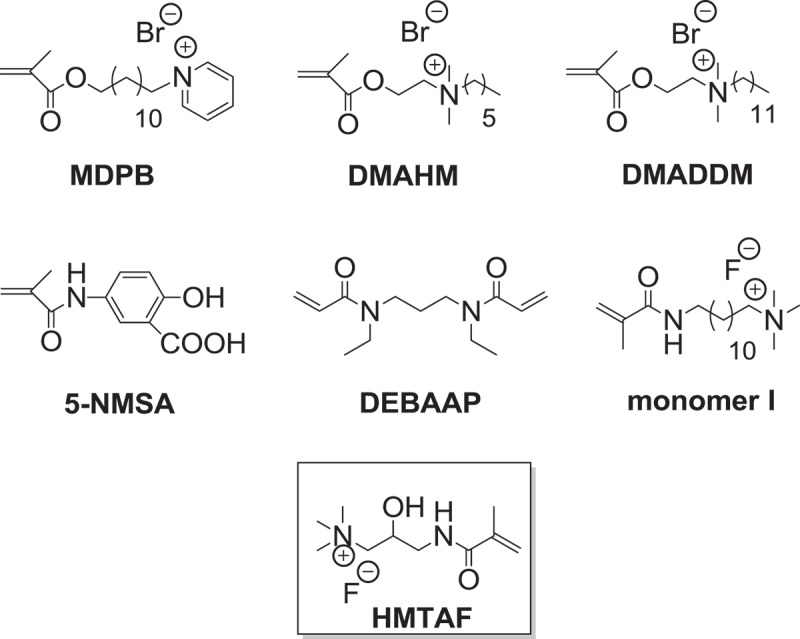
Structures of antibacterial methacrylates: MDPB, DMAHM and DMADDM, methacrylamides: 5-NMSA, DEBAAP, monomer I and HMTAF.

Quaternary ammonium compounds (QACs) are broad-spectrum antibacterial agents with low toxicity [13,14]. They have a wide variety of applications including incorporation in resin adhesives to prevent secondary caries. 12-Methacryloyloxydodecyl pyridinium bromide (MDPB) (Figure 2) is the only antimicrobial monomer being used in a commercial resin adhesive (Clearfil Protect Bond, Kuraray Co., Ltd., Tokyo, Japan). MDPB is a quaternary ammonium monomethacrylate with positively charged pyridinium group [15]. The positive charges could attract the negative charges of bacterial cell wall resulting in cell lysis. However, this monomer still undergoes hydrolysis. Significant reduction of resin-dentin microtensile bond strength after *in vitro* 12-month storage was observed [16]. Therefore, there are still areas to explore new antimicrobial monomers that can effectively bond to tooth structure and withstand hydrolytic and enzymatic degradation for durable bond. Quaternary ammonium methacrylate monomers were synthesized and tested for their antibactierial and mechanical properties, such as *N, N*-dimethylaminohexylmethacrylate (DMAHM) and dimethylaminododecylmethacrylate (DMADDM) (Figure 2) [17]. There are a few literature precedents on (meth)acrylamide-based resin adhesives, mostly without antibacterial moiety, such as 5-NMSA (*N*-methacryloyl-5-aminosalicylic acid) [18] and DEBAAP (*N, N*’-diethyl-1,3-bis(acrylamido)-propane) [9] (Figure 2). 5-NMSA has salicylic acid functional group for binding to calcium, and amide bond which can form hydrogen bonding with amides of collagen. In 2012, Xu and coworkers reported antibacterial fluoride-releasing methacrylamide monomer containing quaternary ammonium group (monomer I) (Figure 2) [19]. However, quaternary ammonium (meth)acrylamide with specific functionalities designed for dentin primers has not widely been synthesized. The objectives of this work were to synthesize and characterize a new quaternary ammonium fluoride methacrylamide-based monomer, **HMTAF**, containing hydrophilic hydroxyl and amide groups for hydrogen bonding to dentin collagen. Hydroxyl group also increases monomer hydrophilicity for better penetration into water-saturated dentin and sufficient resin-dentin bond. The desirable properties of hydrolytic resistance, polymerization capability of methacrylamide group and cytotoxicity of the monomer were also proved.

## Experimental

### General information

Unless otherwise stated, all reactions were performed under a nitrogen atmosphere in oven- or flamed-dried glassware. Please see synthesis and characterization data of each compound in Supplementary Data). Solvents were used as received from suppliers or distilled prior to use following standard procedures. All other reagents were obtained from commercial sources and used without further purification. Column chromatography was performed on SiliaFlash® G60 Silica (60–200 μm, Silicycle). Thin-layer chromatography (TLC) was performed on SiliaPlate^TM^ R10011B-323 (Silicycle) or Silica gel 60 F_254_ (Merck). H_1_, and C_13_ NMR spectroscopic data were recorded on a 300 MHz Bruker FT-NMR Ultra Shield spectrometer using tetramethylsilane as an internal standard. Infrared (IR) spectra were recorded on a Perkin Elmer 783 FTS165 FT-IR spectrometer (Bruker Corp., Billerica, MA, USA). High-resolution ESI mass spectra were obtained on a liquid chromatograph-mass spectrometer (2690, LCT, Waters, Micromass). Gel permeation chromatography (GPC) was performed using an Agilent Technologies 1260 Infinity II (for MDPB polymers) or PL-GPC 220 (for HMTAF polymers) equipped with two PLgel 10 μm MIXED-B columns (Agilent, for MDPB polymers) or two PLgel 5 μm MIXED-C columns (Agilent, for HMTAF polymers) and a guard column (Agilent). Tetrahydrofuran (THF) was used as the eluent at a flow rate of 1 mL/min at 40 °C. A refractive index detector was used and the molecular weights were calibrated against twelve linear polystyrene standards ranging from 162 to 364,000 (Mp[g/mol]).

## Study of hydrolytic stability of HMTAF, MDPB and their polymers

First, 1 mL of 0.1 molar hydrochloric acid (pH = 1) was added to a solution of 10 mg **HMTAF, MDPB** or their polymers in 50 µL methanol. The resulting solutions were then stored at room temperature for 7 days. After that, methanol was evaporated under reduce pressure, and water was removed by freeze-drying process [1]. H NMR spectra of hydrolyzed **HMTAF** and **MDPB** were observed. GPC traces of **HMTAF** and **MDPB** polymers before and after hydrolysis were collected.

## Photopolymerization of HMTAF and MDPB

50 mg of **HMTAF** or **MDPB** was mixed with photoinitiators: 0.25% camphoquinone and 1% ethyl 4-(dimethylamino)benzoate (EDMAB) in methanol, and dried with a gentle stream of air. The thin film was irradiated with a halogen lamp (Valo LED curing, 1300 mW/cm^2^, Ultradent Products Inc., St Louis, MO, USA) for 15 min. FT-IR spectra of HMTAF before and after photopolymerization were observed.

## Cytotoxicity assays

The non-cancerous monkey kidney fibroblast Vero cell lines (ATCC® CCL-81™) were seeded onto 96-well culture plate at a density of 2.5 × 10^3^ cells/well and incubated overnight at 37 °C in humidified atmosphere containing 5% carbon dioxide. Cells were exposed to 0–200 µM of HMTAF in 0.2% v/v DMSO for 72 h. Doxorubicin treatment was also performed as a positive control. Cytotoxicity of HMTAF from triplicate experiments (N = 9) was determined by MTT assay as method reported in Iawsipo *et al* [20]. Plots of % cell viability *vs* concentration of HMTAF and doxorubicin were observed.

## Results and discussion

**Scheme 2.**
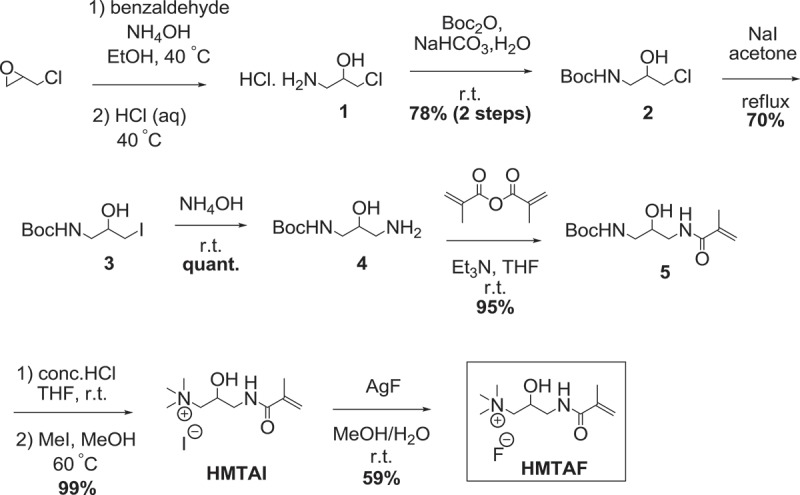
Synthesis of resin adhesive monomer **HMTAF.**

**Scheme 3.**
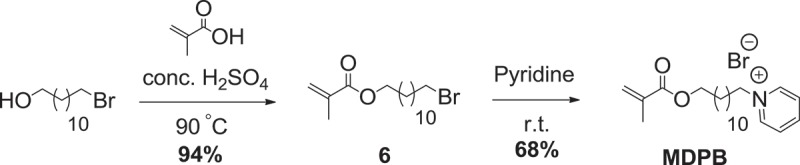
Synthesis of reference monomer **MDPB.**

Resin adhesive monomer **HMTAF** was synthesized in 7 steps as shown in Scheme 2. At first, epoxide ring opening of epichlorohydrin was performed using benzaldehyde and ammonium hydroxide, followed by hydrolysis with hydrochloric acid to give compound **1**[21]. Then amino group of compound **1** was protected with *tert*-butyl carbamate (Boc) to provide 78% of compound **2** over 2 steps. Chlorinated compound **2** was converted to iodinated compound **3** in 70% using sodium iodide. Next, compound **3** was transformed to amino compound **4** by ammonium hydroxide in quantitative yield. Methacrylamide **5** was constructed in 95% yield by reacting amine **4** with methacrylic anhydride. Boc protecting group was removed by concentrated hydrochloric acid, followed by methylation with methyl iodide to provide quaternary ammonium iodide salt **HMTAI** in 99% yield. Finally, iodide ion in **HMTAI** was exchanged with fluoride ion from silver fluoride to yield **HMTAF** in 59%. The overall yield of synthesizing **HMTAF** was 30%. Synthesis of the reference monomer, **MDPB**, the only commercial antibacterial resin adhesive, was performed as shown in Scheme 3. Condensation of 12-bromoundecanol with methacrylic acid and catalytic sulfuric acid was carried out to afford methacrylate **6** in 94%. Substitution of compound **6** with pyridine afforded **MDPB** in 68%.

**Figure 3.**
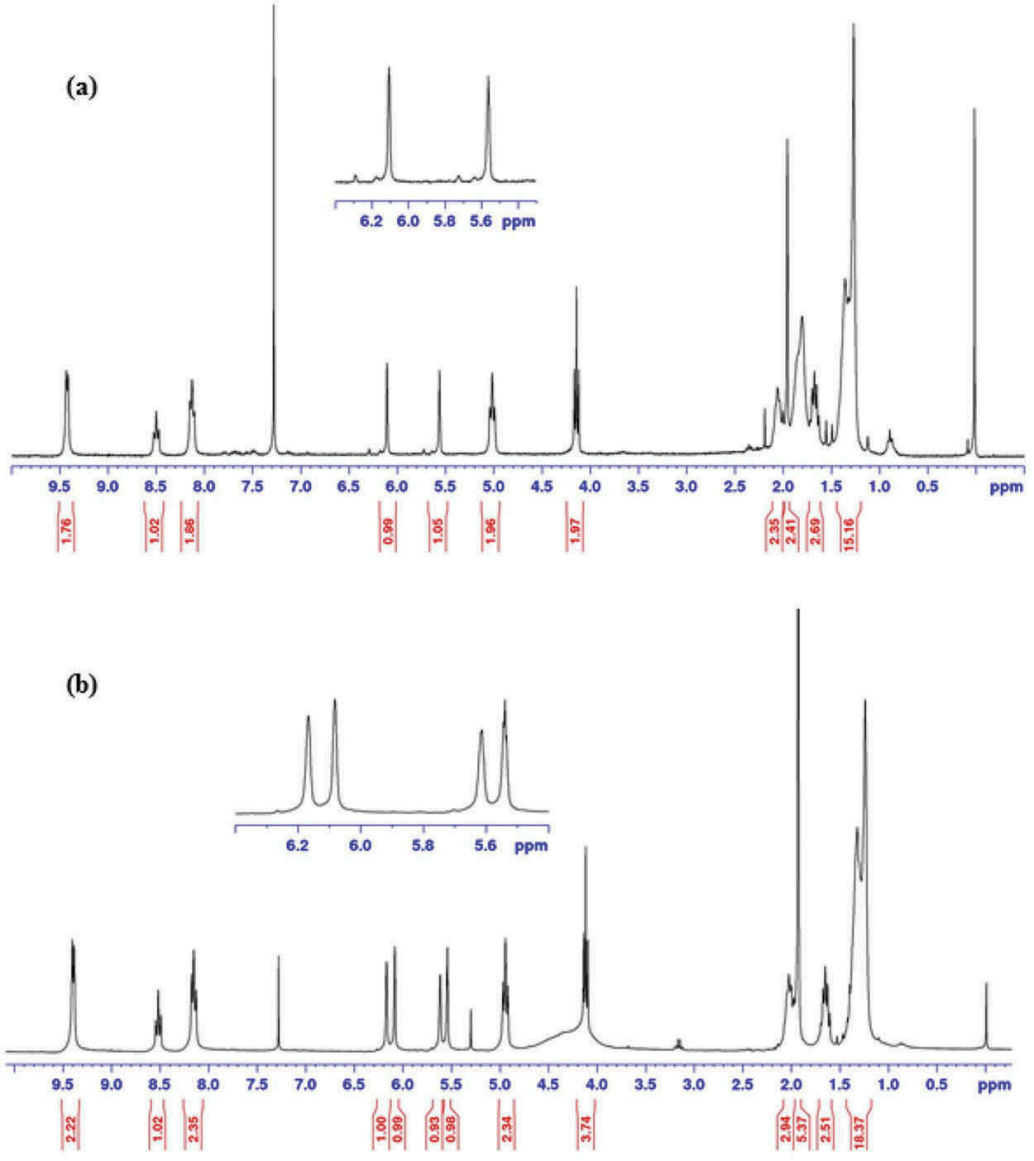
^1^H NMR spectra of **MDPB** (a) before and (b) after treatment with aqueous hydrochloric acid (0.1 M, pH = 1).

**Scheme 4.**
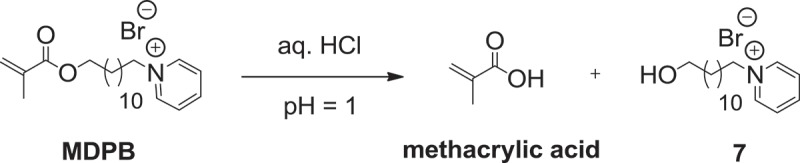
Hydrolytic degradation reaction of **MDPB** in acidic condition.

In order to study the hydrolytic resistance of synthesized monomers, **HMTAF** and the reference monomer, **MDPB**, were individually subjected to an acidic aqueous solution of hydrochloric acid (0.1 M, pH = 1) for a week. H_1_ NMR spectra of **HMTAF** before and after treating with the acid were identical indicating the high stability of monomer **HMTAF** in an acidic environment. In other words, **HMTAF** is a hydrolytic resistant monomer. In contrast, H_1_ NMR spectra of **MDPB** after acidic treatment showed extra peaks of methacrylic acid at 6.17 and 5.61 ppm (Figure 3) resulting from hydrolysis of ester bond of **MDPB** into methacrylic acid and pyridinium bromide salt **7** as shown in Scheme 4. It can be concluded from this experiment that **MDPB** underwent hydrolytic degradation faster than **HMTAF** in an acidic aqueous environment. Exposure of monomers in an acidic aqueous solution exaggerates aging condition via hydrolysis in the oral environment where resin adhesives are periodically exposed to acids from foods and cariogenic bacteria [4,22]. Instability of **MDPB** in a long-term water storage has been well studied by De Munck *et al* [23].

**Figure 4.**
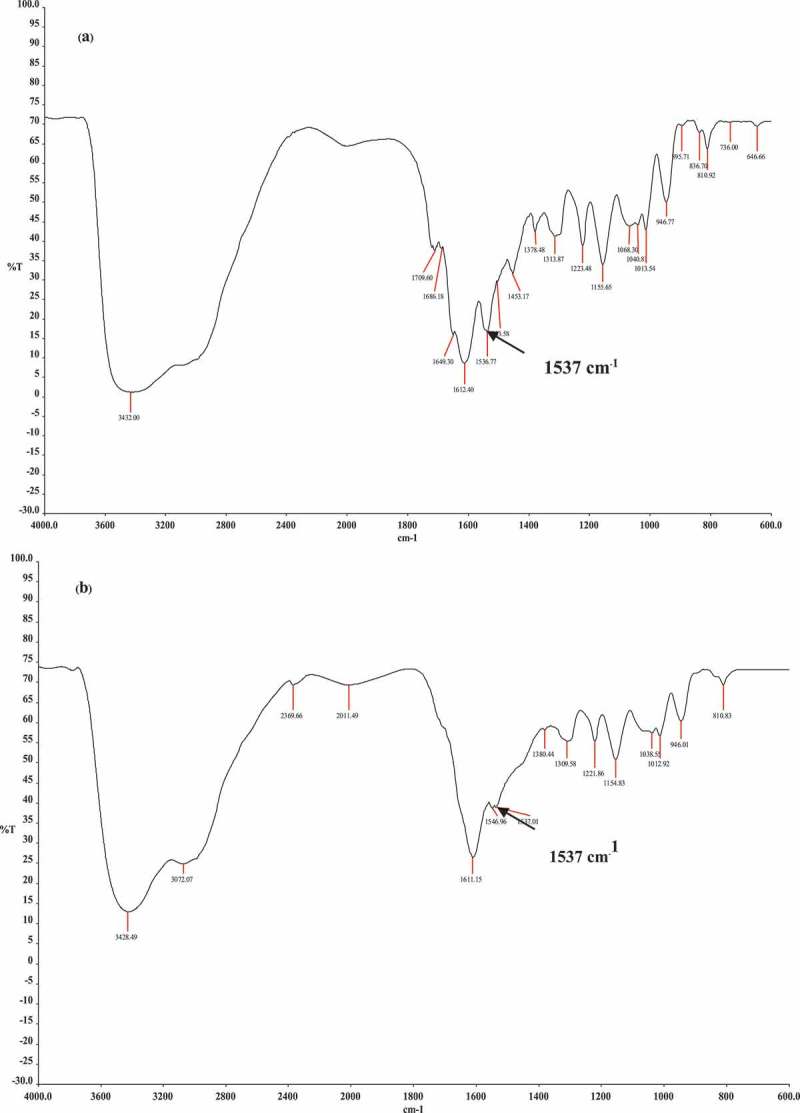
FT-IR spectra of **HMTAF** (a) before and (b) after photopolymerization by curing with a halogen lamp.

After blending **HMTAF** with photoinitiators: camphoquinone and ethyl 4-(dimethylamino)benzoate (EDMAB), and curing with a halogen lamp (blue light), conversion of monomer **HMTAF** into polymer was indicated by significant decrease of intensity of characteristic peak of carbon-carbon double bond at 1537 cm^−1^ in IR spectra as shown in Figure 4 [24]. As a result, monomer **HMTAF** is able to polymerize under the blue light which is used in dental clinics. GPC data of **HMTAF** polymer showed Mw = 8468 g/mol, Mn = 3249 g/mol and PDI = 2.61. Polymers of **HMTAF** and **MDPB** were also subjected to hydrolysis in the same condition as above. After hydrolysis, GPC data showing Mw = 8744 g/mol, Mn = 3226 g/mol and PDI = 2.71 indicated that **HMTAF** polymer did not hydrolyzed in acidic solution within a week. MDPB polymer before hydrolysis gave Mw = 51,881 g/mol, Mn = 25,667 g/mol and PDI = 2.02, but it was partially degraded into oligomers under this condition, Mw = 37,973 g/mol, Mn = 22,171 g/mol, PDI = 1.71 (see all GPC traces in supplementary data).

**Figure 5.**
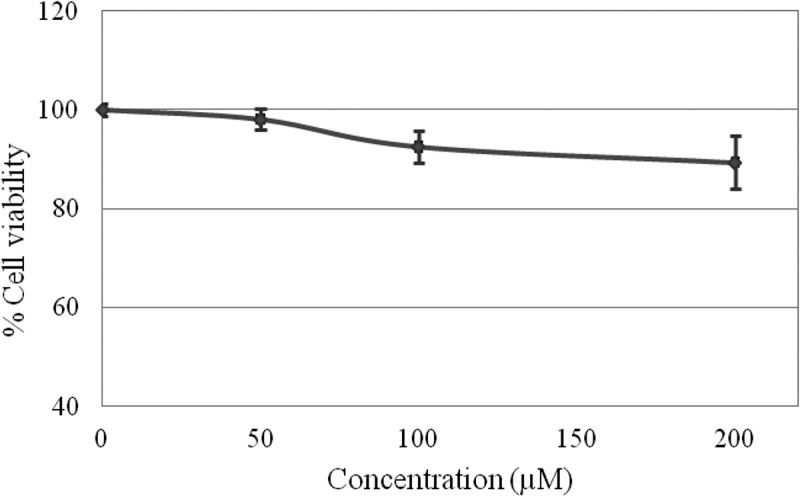
Plot of % cell viability *vs* concentration (µM) of **HMTAF.**

**HMTAF** was non-cytotoxic against Vero cells (non-cancerous African green monkey *Cercopithecus aethiops* kidney fibroblast cell lines). It did not show significant inhibitory effect on Vero growth even at high concentration of 200 µM, since 90% of Vero cells were viable as shown in Figure 5, whereas the positive control, doxorubicin showed IC_50_ value at 1.5 µM (see supplementary data).

## Conclusions

New resin adhesive monomer, **HMTAF**, was successfully synthesized in 30% overall yield. The methacrylamide **HMTAF** and its polymer were stable under an acidic solution whereas the commercial antibacterial methacrylate **MDPB** and its polymer were partially hydrolyzed under the same condition. **HMTAF** could be polymerized by curing with a halogen lamp. It was non-cytotoxic to Vero cell lines. Antibacterial properties of **HMTAF** against *Streptococcus mutans* and *Enterococcus faecalis*, associated with caries formation, will be further investigated. **HMTAF** is a potential monomer to be incorporated into resin adhesives for improving resin-dentin bond durability through hydrolytic resistance in the oral environment and antibacterial activity. The synthesis of **HMTAF** could be improved using a shorter process to enhance the overall yield. Moreover, the phosphate group maybe incorporated into the molecule for bonding to hydroxyapatite in dentin.

## References

[1] Tyas MJ. Placement and replacement of restorations by selected practitioners. Aust Dent J. 2005;50:81–89. 16050086 10.1111/j.1834-7819.2005.tb00345.x

[2] Rho YJ, Namgung C, Jin BH, et al Longevity of direct restorations in stress-bearing posterior cavities: a retrospective study. Oper Dent. 2013;38:572–582. 23550914 10.2341/12-432-C

[3] Peumans M, De Munck J, Van Landuyt KL, et al A 13-year clinical evaluation of two three-step etch-and-rinse adhesives in non-carious class-V lesions. Clin Oral Investig. 2012;16:129–137. 10.1007/s00784-010-0481-z 20931252

[4] Bourbia M, Finer Y. Biochemical stability and interactions of dental resin composite and adhesives with host and bacteria in the oral cavity: a review. J Can Dent Assoc. 2018;84:1–7. 29513214

[5] Demarco FF, Correa MB, Cenci MS, et al Longevity of posterior composite restoration. Dent Mater. 2012;93:87–101. 10.1016/j.dental.2011.09.003 22192253

[6] Opdam NJ, van de Dande FH, Bronkhorst E, et al Longevity of posterior restorations: a systematic review and meta-analysis. J Dent Res. 2014;93:943–949. 25048250 10.1177/0022034514544217 PMC4293707

[7] Breschi L, Maravic T, Cunha SR, et al Dentin bonding systems: from dentin collagen structure to bond preservation and clinical applications. Dent Mater. 2018;34:78–96. 29179971 10.1016/j.dental.2017.11.005

[8] Pashley DH, Tay FR, Breschi L, et al State of the art etch-and-rinse adhesives. Dent Mater. 2011;27:1–16. 21112620 10.1016/j.dental.2010.10.016 PMC3857593

[9] Salz U, Zimmerman J, Zeuner F, et al Hydrolytic stability of self-etching adhesive systems. J Adhes Dent. 2005;7:107–116. 16052759

[10] Al Hiyasat AS, Darmani H, Elbetieha AM. Effects of bisphenol A on adult male mouse fertility. Eur J Oral Sci. 2002;110:163–167. 12013561 10.1034/j.1600-0722.2002.11201.x

[11] Paranjpe A, Bordador LC, Wang MY, et al Resin monomer 2-hydroxyethyl methacrylate (HEMA) is a potent inducer of apoptotic cell death in human and mouse cells. J Dent Res. 2005;84:172–177. 15668336 10.1177/154405910508400212

[12] Geurtsen W Biocompatibility of resin-modified filling materials. Crit Rev Oral Biol Med. 2000;11:333–355. 11021634 10.1177/10454411000110030401

[13] Antonucci JM, Zeiger DN, Tang K, et al Synthesis and characterization of dimethacrylate containing quaternary ammonium functionalities for dental application. Dent Mater. 2012;28:219–228. 22035983 10.1016/j.dental.2011.10.004 PMC3259208

[14] Gerba CP Quaternary ammonium biocides: efficacy in application. Appl Environ Microbiol. 2015;81:464–469. 25362069 10.1128/AEM.02633-14 PMC4277564

[15] Imazato S, Russell RR, McCabe JF Antibacterial activity of MDPB polymer incorporated in dental resin. J Dent. 1995;23:177–181. 7782530 10.1016/0300-5712(95)93576-n

[16] Pupo YM, Farago PV, Nadal JM, et al Effect of a novel quaternary ammonium methacrylate polymer (QAMP) on adhesion and antibacterial properties of dental adhesives. Int J Mol Sci. 2014;15:8998–9015. 24853131 10.3390/ijms15058998 PMC4057771

[17] Cocco AR, Rosa WL, Silva AF, et al A systematic review about antibacterial monomers used in dental adhesive systems: current status and further prospects. Dent Mater. 2015;31:1345–1362. 26345999 10.1016/j.dental.2015.08.155

[18] Nishiyama N, Suzuki K, Asakura T, et al Adhesion of *N*-methacryloyl-ω-amino acid primers to collagen analyzed by ^13^C NMR. J Dent Res. 2001;80:855–859. 11379884 10.1177/00220345010800030201

[19] Xu X, Wang Y, Liao S, et al Synthesis and characterization of antibacterial dental monomers and composites. J Biomed Mater Res Part B. 2012;100:1151–1162. 10.1002/jbm.b.32683 PMC3407682 22447582

[20] Panata I, Ekaruth S, Mathurose P, et al Cytotoxic effects of *Etlingera pavieana* rhizome on various cancer cells and identification of a potential anti-tumor component. J Food Biochem. 2018;42:e12540.

[21] Perrault WR, Pearlman BA, Godrej DB, et al The synthesis of *N*-aryl-5(*S*)-aminomethyl-2-oxazolidinone antibacterials and derivatives in one step from aryl carbamates. Org Proc Res Dev. 2003;7:533–546.

[22] Pinna R, Usai P, Filigheddu E, et al The role of adhesive materials and oral biofilm in the failure of adhesive resin restorations. Am J Dent. 2017;30:285–292. 29178733

[23] De Munck J, Mine A, Vivan Cardoso M, et al Effect of dentin location and long-term water storage on bonding effectiveness of dentin adhesives. Dent MaterJ. 2011;30:7–13. 21282894 10.4012/dmj.2010-085

[24] Nakano Y, Shinke K, Ueno K, et al Solid polymer electrolytes prepared from poly(methacrylamide) derivative having tris(cyanoethoxymethyl) group as its side chain. Solid State Ion. 2016;286:1–6.

