# Supplementary material for: Synthesis and characterization of new hydrolytic-resistant dental resin adhesive monomer HMTAF
Source: Des Monomers Polym. 2019 May 12;22(1):106–13. doi: 10.1080/15685551.2019.1615789 (PMC6522974; doi:10.1080/15685551.2019.1615789)
Supplement: Supplemental Material [file TDMP_A_1615789_SM0463.pdf]

Supplementary Data

Synthesis and Characterization of New Hydrolytic Resistant Dental Resin Adhesive Monomer HMTAF

Nattawut Decha[1], Supitcha Talungchit[2], Panata Iawsipo[3], Arthit Pikulngam[1], Piangkwan Saiprasert[2] and Chittreeya Tansakul[1,*]

[1] Department of Chemistry and Center of Excellence for Innovation in Chemistry, Faculty of Science, Prince of Songkla University, Hat Yai, Songkhla 90112, Thailand

[2] Department of Conservative Dentistry, Faculty of Dentistry, Prince of Songkla University, Hat Yai, Songkhla 90112, Thailand

[3] Department of Biochemistry and Center of Excellence for Innovation in Chemistry, Faculty of Science, Burapha University, Chonburi 20000, Thailand

* Corresponding author. Tel.: +66 74 288446; fax: +66 74 558841.
Email address: chittreeya.t@psu.ac.th (C. Tansakul).

List of Supplementary Data

	Page
1. Synthesis and Characterization Data	2
2. GPC traces of **HMTAF** and **MDPB** before and after hydrolysis	7
3. Plot of % cell viability *vs* concentration (μM) of doxorubicin	9
4. Raw data of %cell viability *vs* concentration (μM) of **HMTAF**	9
5. ^{1}H and ^{13}C NMR Spectra	10
6. Reference	15

1. Synthesis and Characterization Data

1.1 Synthesis of resin adhesive monomer HMTAF

1.1.1 Synthesis of ammonium salt 1

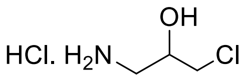

1

Following the procedure of Perrault et al.,[1] to a solution of benzaldehyde (2.70 mL, 21.3 mmol) and epichlorohydrin (2.00 mL, 25.5 mmol) in ethanol (5.30 mL) was added concentrated ammonium hydroxide (17.5 M, 2.25 mL, 38.2 mmol). The reaction mixture was heated at 40 ˚C overnight. The reaction mixture was concentrated under reduced pressure, and toluene (10 mL) was added to the resulting crude oil. Then a solution of hydrochloric acid (11.65 M, 3.40 mL, 39.8 mmol) was added over 5 min. The two-phase mixture was heated at 40 ˚C for 3 hours. The upper phase was extracted with water (3×20 mL). The combined aqueous phase was concentrated under reduced pressure to give 3.56 g of ammonium salt **1** as yellow viscous oil. The crude oil was used without further purification.

1.1.2 Synthesis of Boc protected amine 2

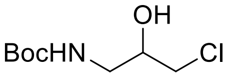

2

A solution of 1-amino-3-chloropropan-2-ol hydrochloride (ammonium salt **1**, 717.7 mg, 4.91 mmol) in methanol (3 mL) was placed into round bottom flask, and a solution of sodium bicarbonate (469 mg, 5.59 mmol) in water (1.7 mL) was added slowly. Then a solution of di-*tert*-butyl dicarbonate (1.23 mL, 5.25 mmol) in methanol (2 mL) was added slowly, and the reaction mixture was stirred at room temperature overnight. Methanol was removed under reduced pressure. Methylene chloride (10 mL) and water (5 mL) were added to the crude oil. The aqueous phase was extracted with methylene chloride (3×8 mL). The combined methylene chloride layer was dried over sodium sulfate, filtered and concentrated under reduced pressure to give 803 mg (78 % yield, over 2 steps) of Boc protected amine **2** as slightly yellow viscous oil. R_f = 0.5 (1:1 hexane:ethyl acetate, *p*-anisaldehyde stain); FT-IR (neat) ν_{max} 3335, 2979, 2934, 1694, 1521, 1253, 1169, 751 cm^{-1}; ^{1}H NMR (300 MHz, CD_3OD) δ 3.80 (qn, *J* = 6.0 Hz, 1H), 3.58 (dd, *J* = 11.4, 6.0 Hz, 1H), 3.49 (dd, *J* = 11.4, 6.0 Hz, 1H), 3.24 (dd, *J* = 13.8, 6.0 Hz, 1H), 3.12 (dd, *J* = 13.8, 6.0 Hz, 1H), 1.44 (s, 9H) ppm; ^{13}C NMR, DEPT (75 MHz, CD_3OD) δ 158.6 (C), 80.3 (C), 71.6 (CH), 47.8 (CH_2), 44.8 (CH_2), 28.7 ($3{\times}CH_3$) ppm; HMRS (ESI)*m/z* calcd for $C_8H_{16}ClNNaO_3^+$ $[M+Na]^+$ 232.0716, found 232.0715.

1.1.3 Synthesis of iodo compound 3

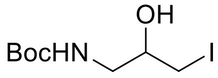

3

A solution of *tert*-butyl 3-chloro-2-hydroxypropylcarbamate (Boc protected amine **2**, 739.6 mg, 3.53 mmol) in anhydrous acetone (35 mL) was placed into round bottom flask, and sodium iodide (5.29 g, 35.3 mmol) was added. The reaction mixture was refluxed overnight. After reaction was completed, acetone was removed under reduced pressure. Then methylene chloride (35 mL) was added into the crude product, and washed with water (3×35 mL). The methylene chloride layer was dried over sodium sulfate, filtered and concentrated under reduced pressure to give 737 mg (70% yield) of iodo compound **3** as yellow viscous oil. $R_f = 0.5$ (1:1 hexane:ethyl acetate, *p*-anisaldehyde stain); FT-IR (neat) ν_{max} 3338, 2978, 1686, 1523, 1252, 1169 cm^{-1}, ^{1}H NMR (300 MHz, CD_3OD) δ 3.83 (qn, *J* = 6.0 Hz, 1H), 3.59 (dd, *J* = 11.4, 6.0 Hz, 1H), 3.49 (dd, *J*= 11.4, 6.0 Hz, 1H), 3.28-3.10 (m, 2H), 1.45 (s, 9H) ppm; ^{13}C NMR, DEPT (75 MHz, CD_3OD): δ 158.6 (C), 80.3 (C), 71.3 (CH), 46.8 (CH_2), 28.7 (3×CH_3), 10.3 (CH_2) ppm; HMRS (ESI) *m/z* calcd for $C_8H_{16}INNaO_3^+$ $[M+Na]^+$ 324.0073, found 324.0071.

1.1.4 Synthesis of amine 4

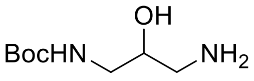

4

Tert-butyl(2-hydroxyiodopropyl)carba- mate (iodo compound **3**, 530 mg, 1.76 mmol) was placed into round bottom flask, and concentrated ammonium hydroxide (17.5 M, 8 mL, 140 mmol) was added. The reaction mixture was stirred at room temperature until it turned to homogeneous mixture. After that excess of ammonium hydroxide was removed under reduced pressure to give 700 mg (quantitative yield) of amine **4** as yellow viscous oil. FT-IR (neat) ν_{max} 3338, 2978, 1686, 1523, 1252, 1169 cm^{-1}; ^{1}H NMR (300 MHz, CD_3OD) δ 3.68-3.60 (m, 1H), 3.10 (d, *J* = 6.0 Hz, 2H), 3.08 (dd, *J* = 12.9, 6.0 Hz, 1H), 2.78 (dd, *J* = 12.9, 6.0 Hz, 1H), 1.42 (s, 9H) ppm; ^{13}C NMR, DEPT (75 MHz, CD_3OD) δ 158.9 (C), 80.7 (C), 68.3 (CH), 44.9 (CH_2), 43.8 (CH_2), 28.7 (3×CH_3); HMRS (ESI) *m/z* calcd for $C_8H_{16}INNaO_3^+$ $[M+Na]^+$ 213.1215, found 213.1215.

1.1.5 Synthesis of compound 5

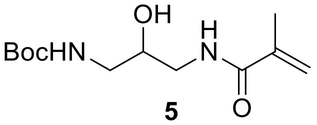

5

Triethylamine (96 µL, 0.69 mmol) was added to a solution of *tert*-butyl (3-amino-2-hydroxypropyl)carbamate (amine **4**, 130.5 mg, 0.69 mmol) in anhydrous THF (69 mL). Then the mixture was cooled to 0°C and methacrylic anhydride (102 µL, 0.69 mmol) was added slowly. The reaction mixture was stirred for 5 h. THF in the resulting solution was removed under reduced pressure. Methylene chloride (60 mL) was added to the crude oil, and washed with saturated $NaHCO_3$ (3×60mL). The methylene chloride layer was dried over sodium sulfate, filtered and concentrated under reduced pressure to give yellow viscous oil. The crude oil was purified by column chromatography with 4:1 hexane:ethyl acetate, followed by 100% ethyl acetate to give 169 mg (95% yield) of methacrylamide **5** as colorless viscous oil of. R_f = 0.63 (100 % ethyl acetate, UV, *p*-anisaldehyde stain); FT-IR (neat) ν_{max} 3338, 2978, 2932, 1691, 1618, 1534, 1252 cm^{-1}; ^1H NMR (300 MHz, acetone-d_6) δ 7.40 (brs, 1H), 6.07 (brs, 1H), 5.75 (s, 1H), 5.34 (t, J = 1.5 Hz, 1H), 4.35 (d, J = 5.4 Hz, 1H), 3.70 (t, J = 5.4 Hz, 1H), 3.34-3.29 (m, 2H), 3.14-3.09 (m, 2H), 1.93 (s, 3H), 1.40 (s, 9H) ppm; ^{13}C NMR, DEPT (75 MHz, acetone-d_6): δ 168.6 (C), 156.6 (C), 140.3 (C), 118.8 (CH_2), 78.2 (C), 69.9 (CH), 43.6 (CH_2), 42.7 (CH_2), 27.7 (3×CH_3), 17.9 (CH_3) ppm; HMRS (ESI) *m/z*calcd for $C_{12}H_{22}N_2NaO_4^+$ $[M+Na]^+$ 281.1477, found 281.1477.

1.1.6 Synthesis of HMTAI

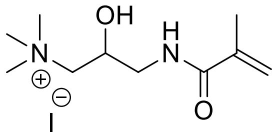

HMTAI

A solution of *tert*-butyl 2-hydroxy-3-(3-methylbuta-1,3-dien-2-ylamino) propylcarbamate (methacrylamide **5**, 447 mg, 1.73 mmol) in THF (17 mL) was placed into round bottom flask, and concentrated hydrochloric acid (283 µL, 3.47 mmol) was added slowly. The reaction mixture was stirred at room temperature for 24 h. The liquid phase was decanted from precipitated product. After that, the precipitate was washed with methylene chloride (10×10 mL), and redissolved in methanol (50 mL). Iodomethane (1.66 mL, 25.9 mmol) was added to the solution. The reaction mixture was heated at 60°C overnight. Methanol was removed under reduced pressure to give 562 mg (99% yield) of **HMTAI** as yellow viscous oil. FT-IR (neat) ν_{max} 3432, 1645, 1612, 1223, 1537, 1155 cm^{-1}; ^1H NMR (300 MHz, CD_3OD) δ 5.82 (s, 1H), 5.46 (t, J = 1.5 Hz, 1H), 4.04-3.96 (m, 1H), 3.41-3.38 (m, 2H), 3.01 (dd, J = 12.9, 6.0 Hz, 1H), 3.01 (s, 9H), 2.86 (dd, J = 12.9, 6.0 Hz, 1H), 1.95 (s, 3H) ppm; ^{13}C NMR, DEPT (75 MHz,CD_3OD): δ 170.5 (C), 139.3 (C), 120.2 (CH_2), 66.5 (CH), 42.9 (CH_2), 42.6 (CH_2), 29.6 (2×CH_3), 17.6 (CH_3), 8.2 (CH_3) ppm; HMRS (ESI) *m/z* calcd for $C_{10}H_{21}N_2NaO_2^+$ $[M+Na]^{2+}$ 224.1495, found 224.1496.

1.1.7 Synthesis of HMTAF

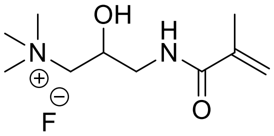

HMTAF

A solution of AgF (15.22 mg, 0.12 mmol) in water (3 mL) was added to a solution of 2-hydroxy-3-methacrylamido-*N*,*N*,*N* trimethylpropan-1-aminium iodide (**HMTAI**, 39.4 mg, 0.12 mmol) in methanol (1 mL) under dimmed light for 10 min. AgI was precipitated, and removed by centrifugation. Methanol was removed under reduced pressure, and water was removed by freeze-drying process to give 15.50 mg (59% yield) of **HMTAF** as a pale yellow viscous oil. FT-IR (neat) ν_{max} 3432, 1645, 1612, 1223, 1537, 1155 cm^{-1}; ^{1}H NMR (300 MHz, CD_3OD) δ 5.77 (s, 1H), 5.42 (s, 1H), 4.01-3.93 (m, 1H), 3.35 (d, *J* = 6.0 Hz, 2H), 3.05 (dd, *J* = 12.9, 6.0 Hz, 1H), 2.99 (s, 9H), 2.83 (dd, *J* = 12.9, 6.0 Hz, 1H), 1.95 (s, 3H) ppm; ^{13}C NMR, DEPT (75 MHz, CD_3OD) δ 170.6 (C), 139.4 (C), 119.8 (CH_2), 66.6 (CH), 42.7 (CH_2), 42.5 (CH_2), 26.1 (CH_3), 17.4 (CH_3) ppm; HRMS (ESI) *m/z* calcd for $C_{10}H_{21}N_2NaO_2^+$ [M+Na]$^{2+}$ 224.1495, found 224.1496.

1.2 Synthesis of reference monomer MDPB

1.2.1 Synthesis of methacrylate 6

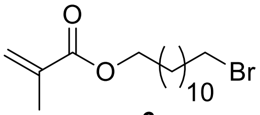

6

To a solution of 12-bromododecanol (100 mg, 0.38 mmol) in methacrylic acid (320 µL, 3.80 mmol) was added concentrated sulfuric acid (4 µL, 0.038 mmol). The reaction mixture was heated to 90 °C, and stirred for 1 hour. The reaction mixture was then cooled to room temperature, and methylene chloride (30 mL) and saturated sodium carbonate (30 mL) were added. The organic layer was separated, and washed with saturated sodium carbonate (2×20 mL), water (20 mL) and with brine (20 mL), dried over sodium sulfate, and concentrated under reduced pressure to give 119 mg (94% yield) of methacrylate **6** as pale yellow oil. FT-IR (neat) ν_{max} 2927, 2854, 1718, 1638, 1455, 1321, 1296, 1166, 939 cm^{-1}; ^{1}H NMR (300 MHz, $CDCl_3$) δ 6.08 (s, 1H), 5.53 (s, 1H), 4.12 (t, *J* = 6.6 Hz, 2H), 3.40 (t, *J* = 6.6 Hz, 2H), 1.93 (s, 3H), 1.84 (p, *J* = 6.9 Hz, 2H), 1.66 (p, *J* = 6.9 Hz, 2H), 1.63-1.26 (m, 16H) ppm; ^{13}C NMR, DEPT δ 167.4 (C), 136.5 (C), 125.0 (CH_2), 64.7 (CH_2), 33.8 (CH_2), 32.8 (CH_2), 29.5 (2×CH_2), 29.4 (CH_2), 29.2 (CH_2), 28.7 (CH_2), 28.6 (CH_2), 28.1 (CH_2), 25.9 (CH_2), 18.3 (CH_3) ppm.

1.2.2 Synthesis of MDPB

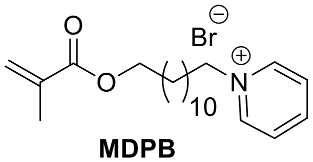

MDPB

12-Bromododecylmethacrylate (methacrylate **6**, 80.3 mg, 0.241 mmol) was dissolved in pyridine (311 µL, 3.86 mmol), and stirred overnight at room temperature. The solution was concentrated under reduced pressure to give crude yellow oil. The crude product was purified by column chromatography with 1:4 methanol:methylene chloride to give 67.8 mg (68% yield) of **MDPB** as pale yellow oil. FT-IR (neat) ν_{max} 2925, 2852, 1711, 1638, 1489, 1467, 1174, 941 cm^{-1}; ^{1}H NMR (300 MHz, $CDCl_3$) δ 9.44 (d, *J* = 6.3 Hz, 2H), 8.49 (t, *J* = 7.8 Hz, 1H), 8.11 (dd, *J* = 6.3, 7.8 Hz, 2H), 6.00 (s, 1H), 5.46 (s, 1H), 4.90 (t, *J* = 6.6 Hz, 2H), 4.03 (t, *J* = 6.6 Hz, 2H), 1.96 (m, 2H), 1.85 (s, 3H), 1.57 (m, 2H), 1.41-1.07 (m, 16H) ppm; ^{13}C NMR, DEPT (75 MHz, $CDCl_3$) δ 167.6 (C), 145.1 (CH), 145.0 (CH), 136.6 (C), 128.4 (3×CH), 125.1 (CH_2), 64.8 (CH_2), 62.3 (CH_2), 31.9 (CH_2), 29.7 (CH_2), 29.4 (CH_2), 29.3 (CH_2), 29.2 (2×CH_2), 29.0 (CH_2), 28.6 (CH_2), 26.0 (CH_2), 25.9 (CH_2), 18.3 (CH_3) ppm.

2. GPC traces of HMTAF and MDPB before and after hydrolysis

2.1 GPC trace of HMTAF before hydrolysis

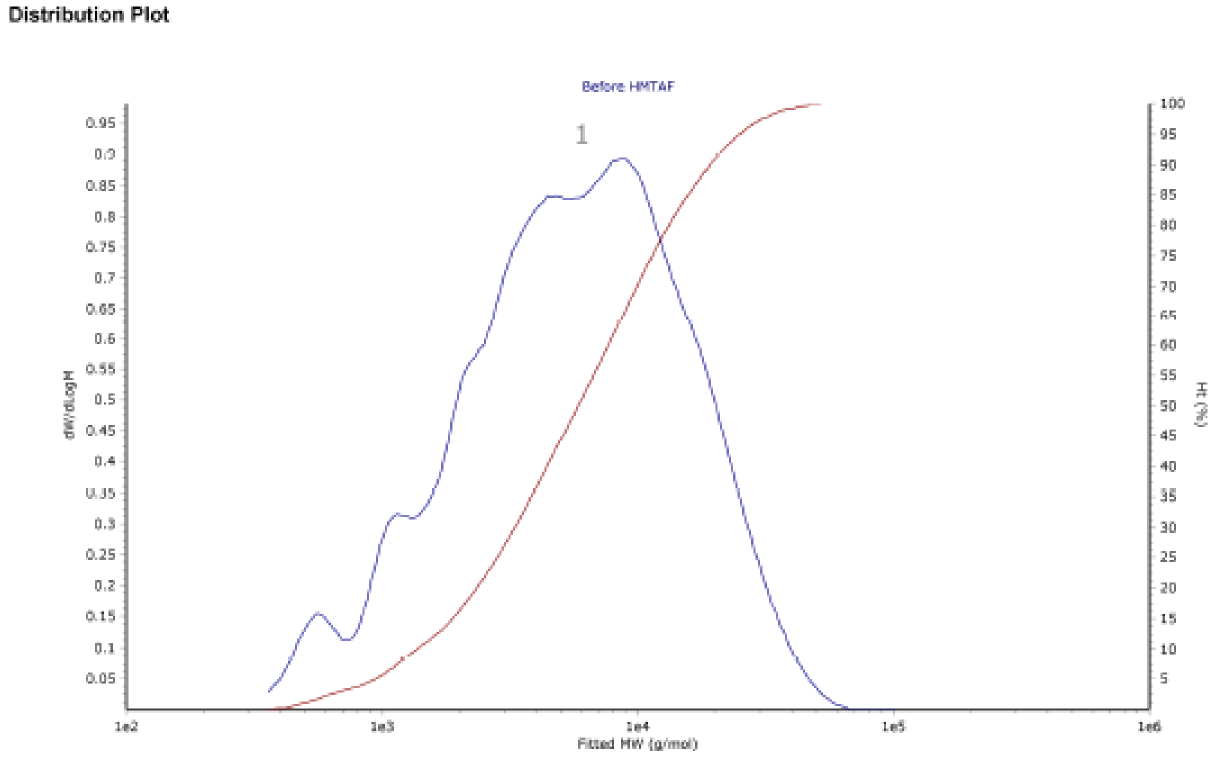

2.2 GPC trace of HMTAF after hydrolysis

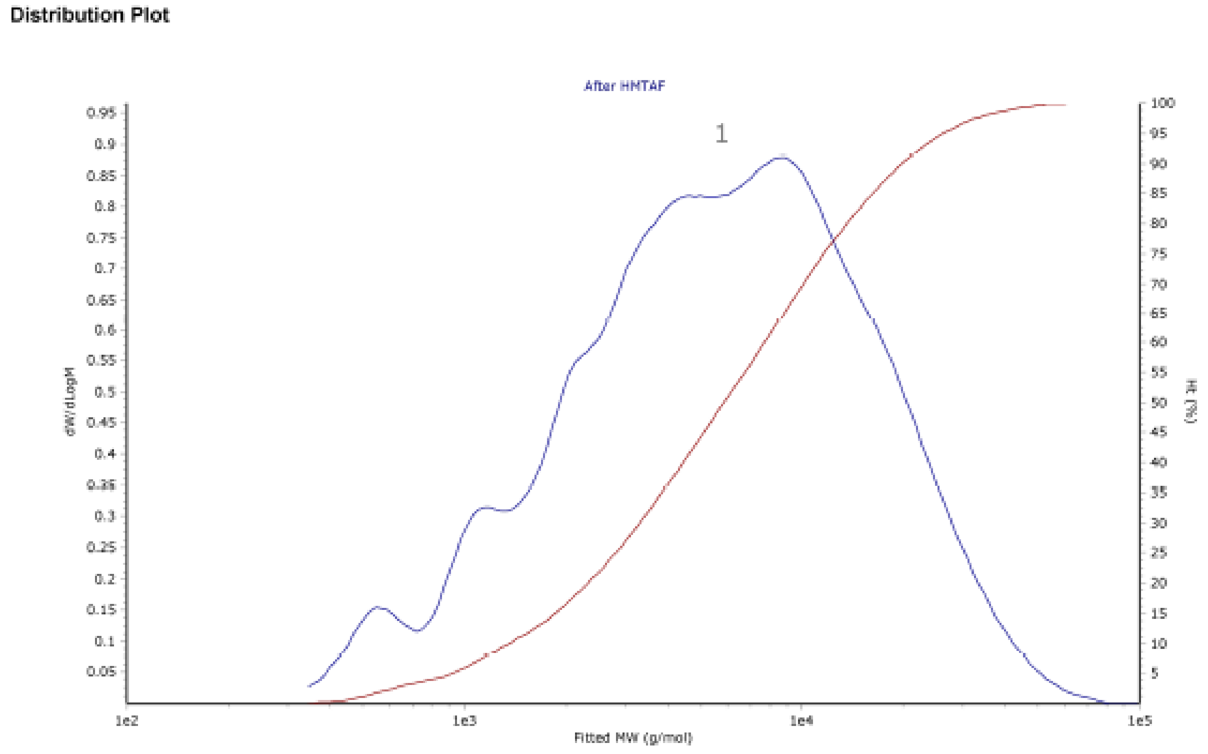

2.3 GPC trace of MDPB before hydrolysis

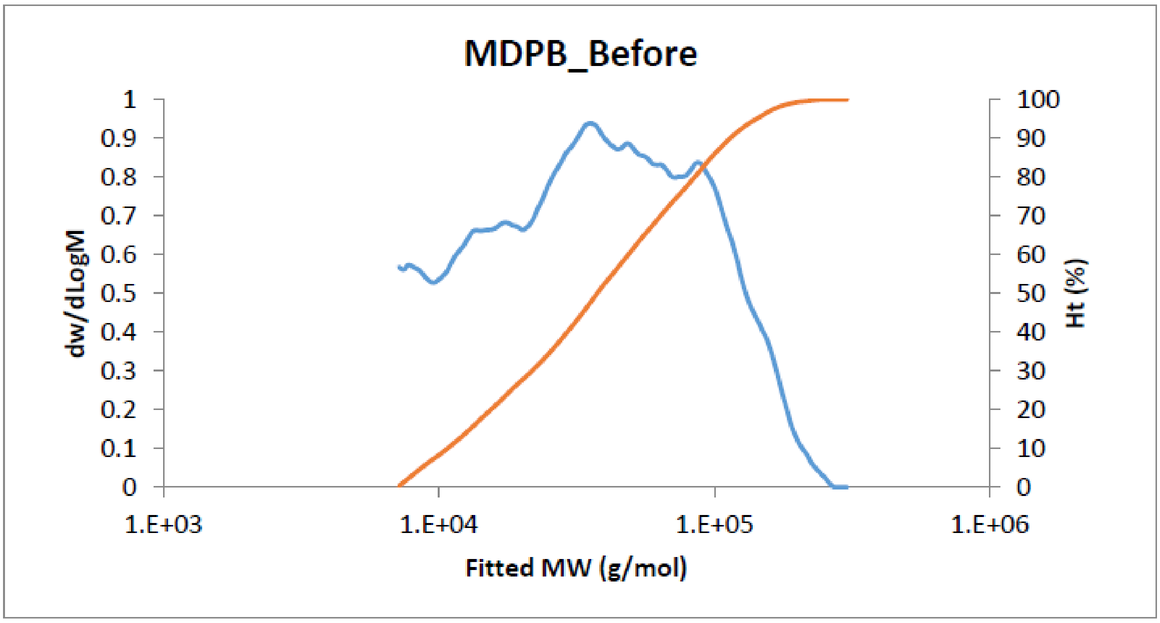

2.4 GPC trace of MDPB after hydrolysis

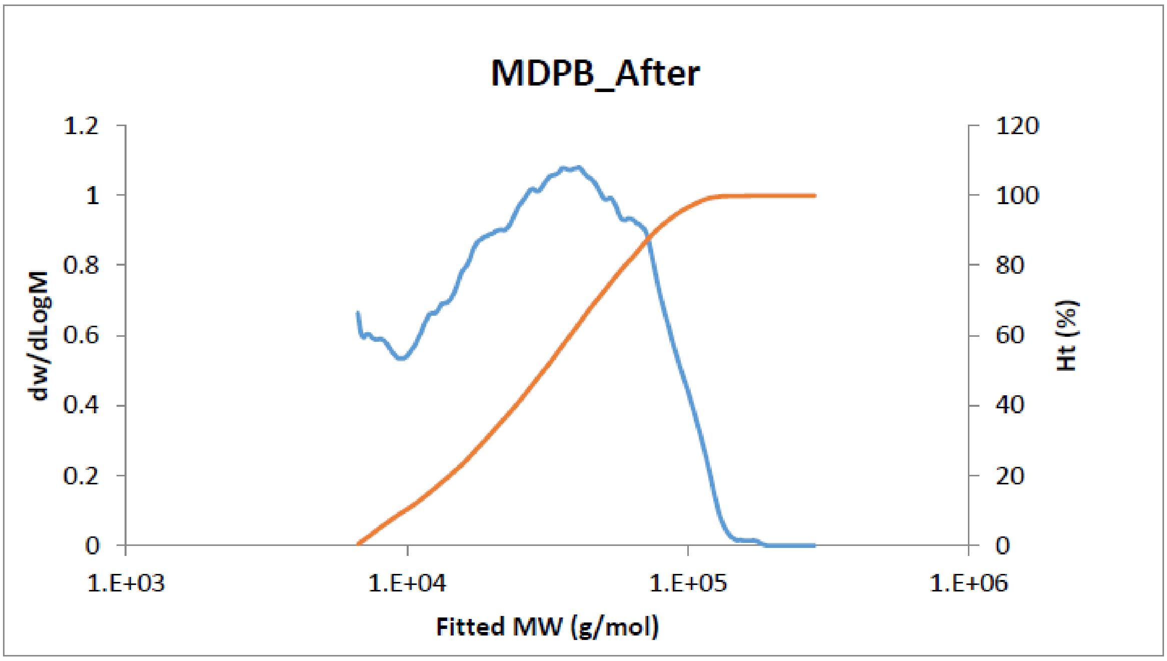

3. Plot of % cell viability *vs* concentration (µM) of doxorubicin (a positive control)

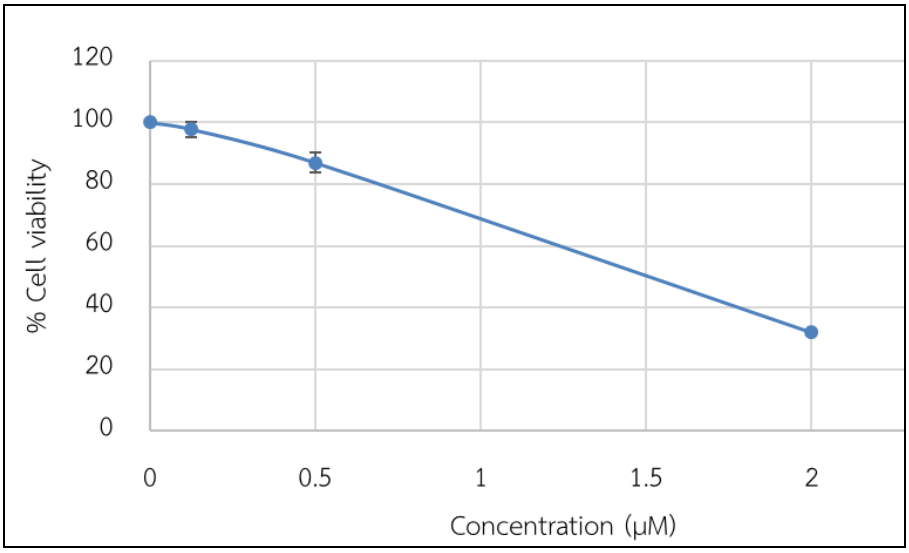

4. Raw data of %cell viability *vs* concentration (µM) of HMTAF

concentration (µM)	% cell viability	S.D.
0	100	1.22
50	98.07	2.14
100	92.53	3.25
200	89.34	5.36

5. ^{1}H and ^{13}C NMR Spectra

^{1}H (300 MHz) NMR spectrum of compound **1** in CD_3OD

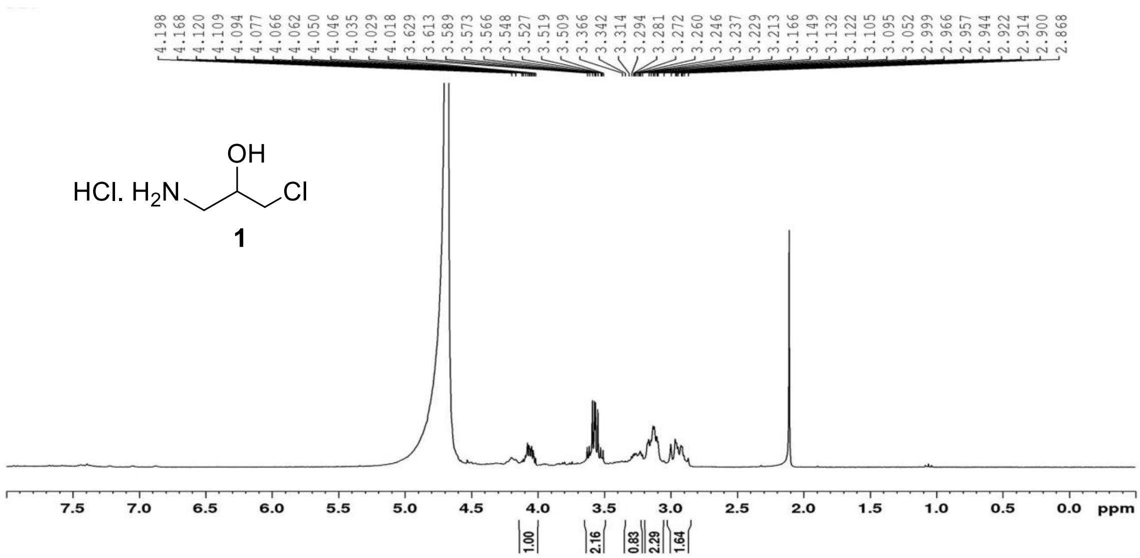

^{1}H (300 MHz) NMR spectrum of compound **2** in CD_3OD

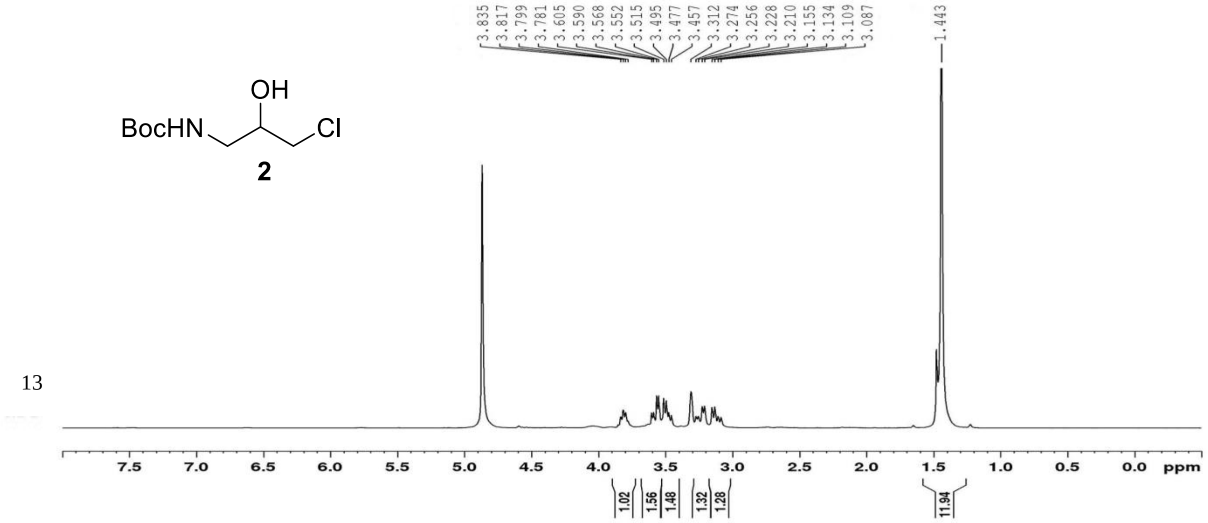

13

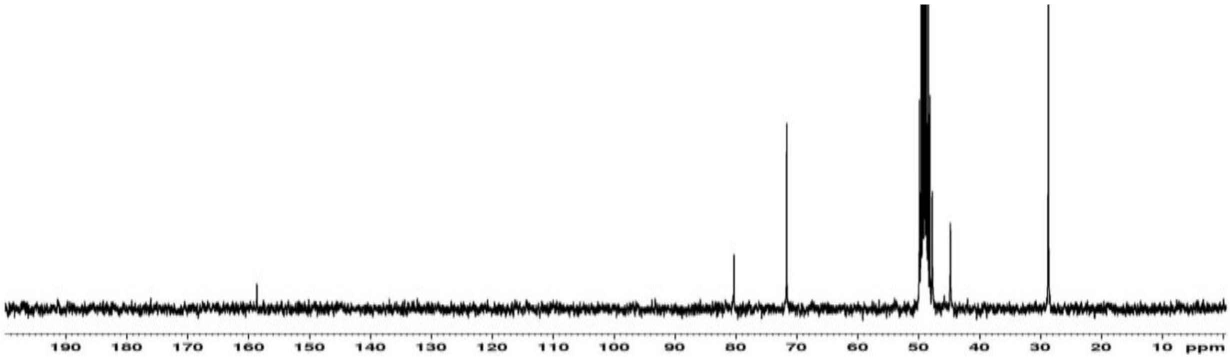

^{1}H (300 MHz) NMR spectrum of compound **3** in CD_3OD

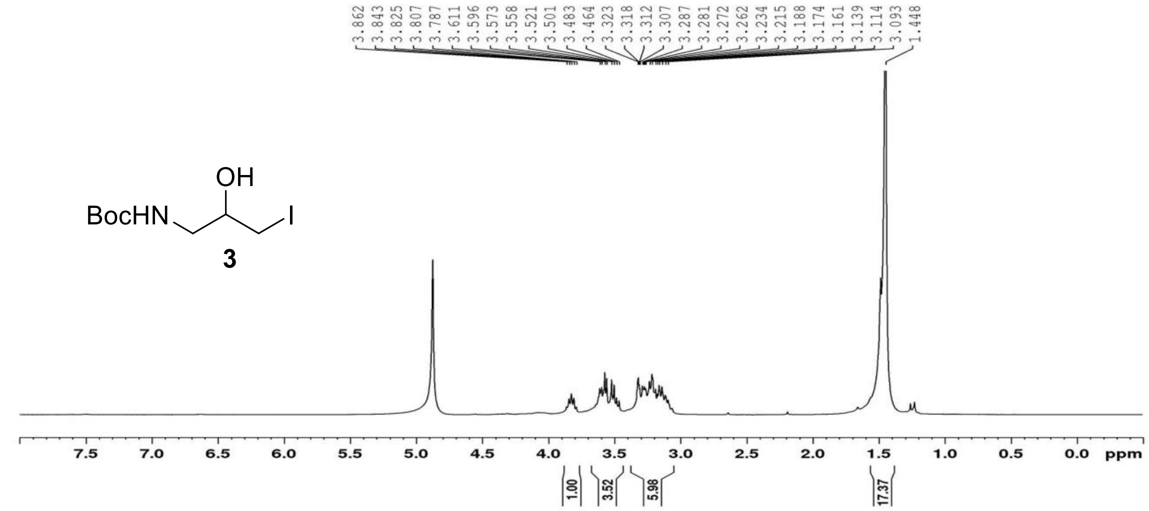

^{13}C (75 MHz) NMR spectra of compound **3** in CD_3OD

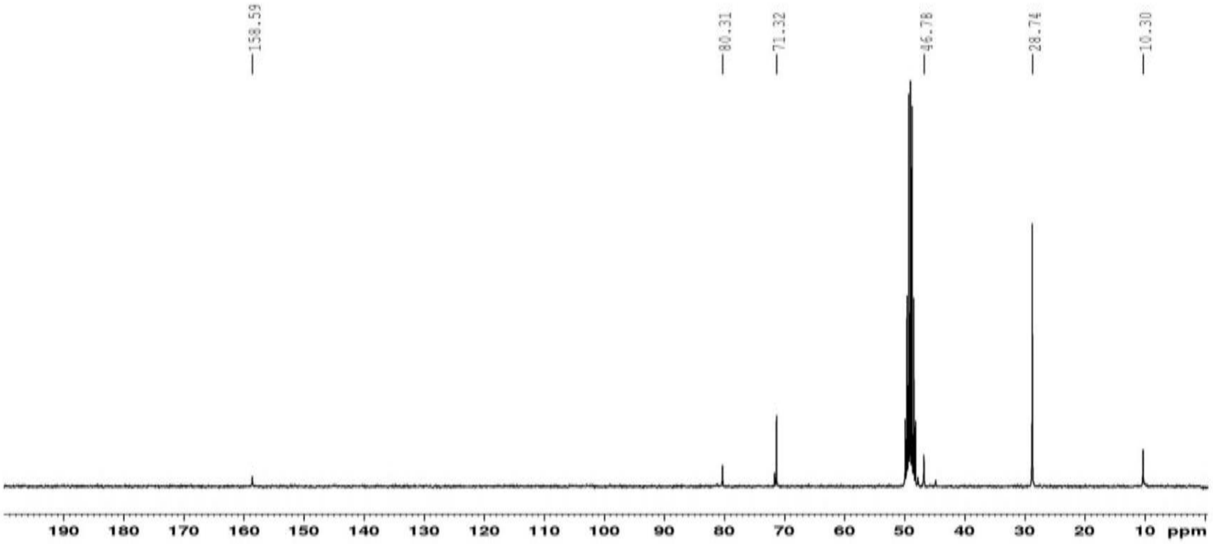

^{1}H (300 MHz) NMR spectrum of compound **4** in CD_3OD

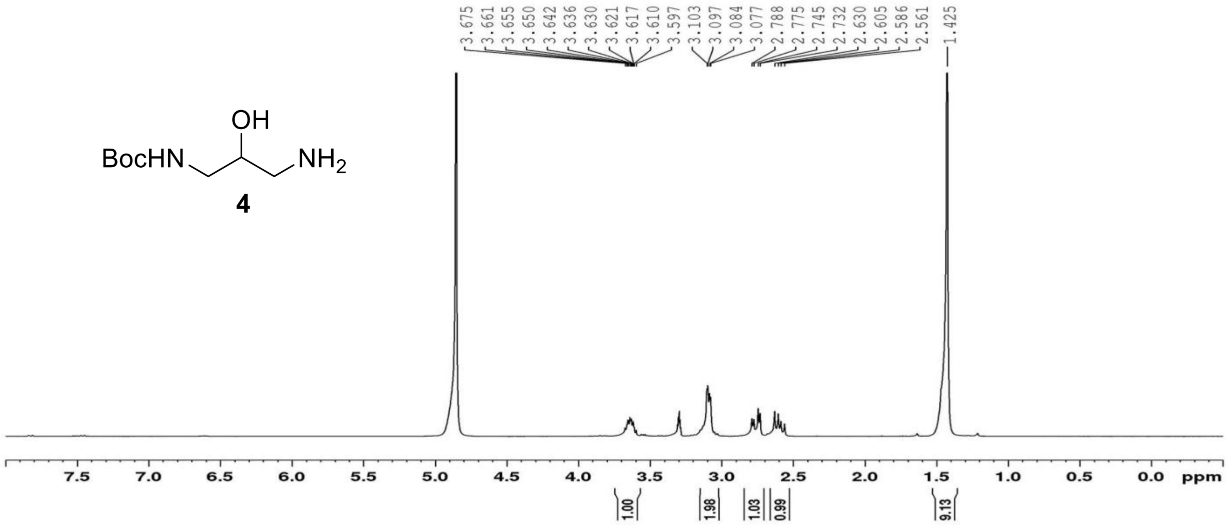

^{13}C (75 MHz) NMR spectra of compound **4** in CD_3OD

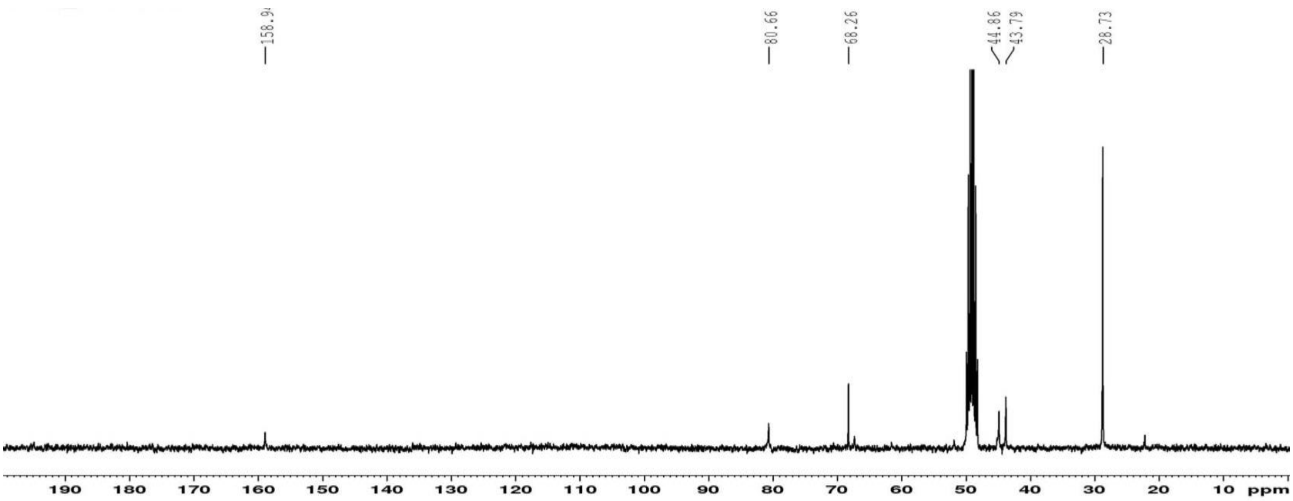

^{1}H (300 MHz) NMR spectrum of compound **5** in acetone-d_6

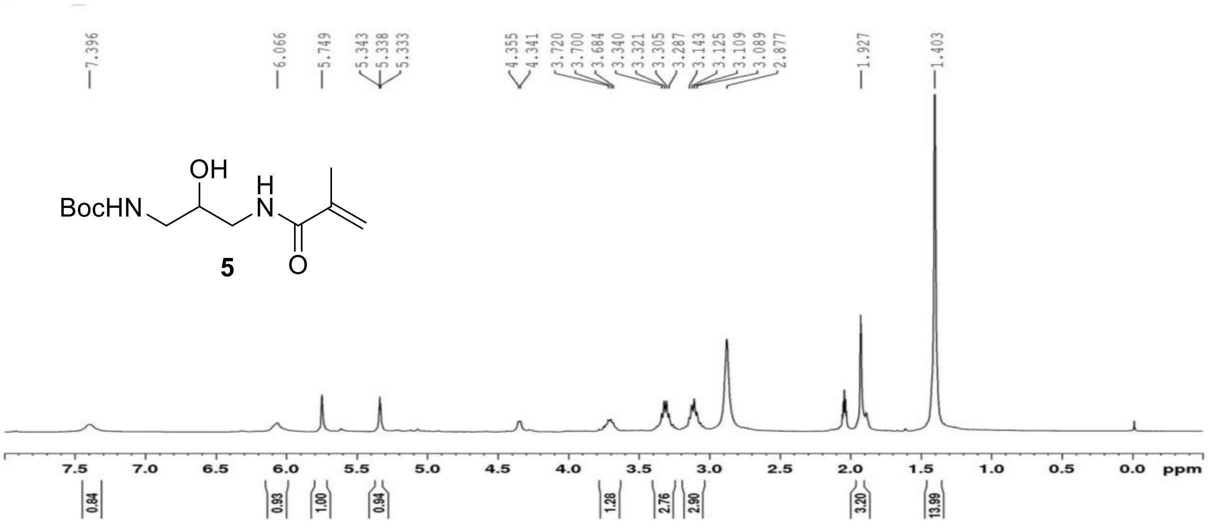

^{13}C (75 MHz) NMR spectra of compound **5** in acetone-d_6

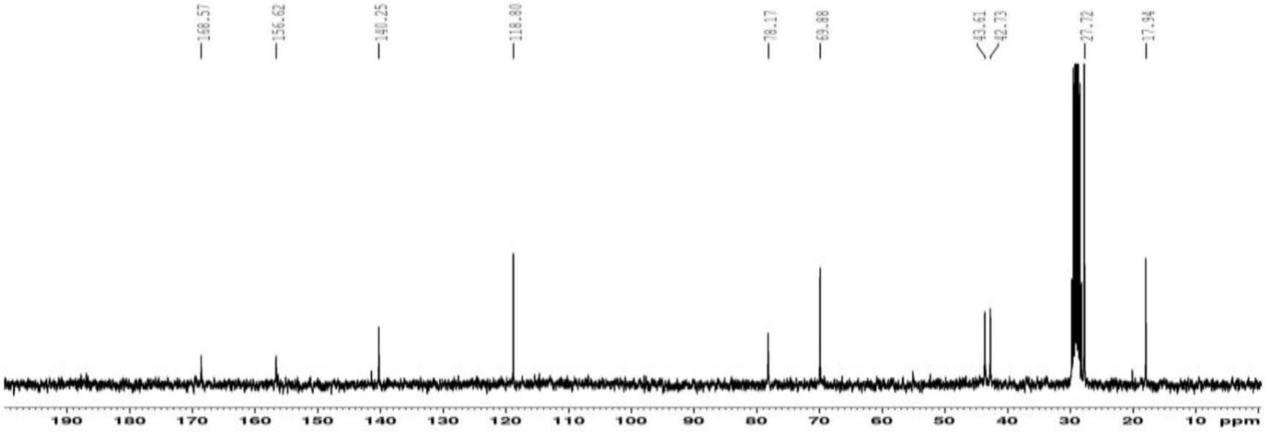

^{1}H (300 MHz) NMR spectrum of **HMTAI** in CD_3OD

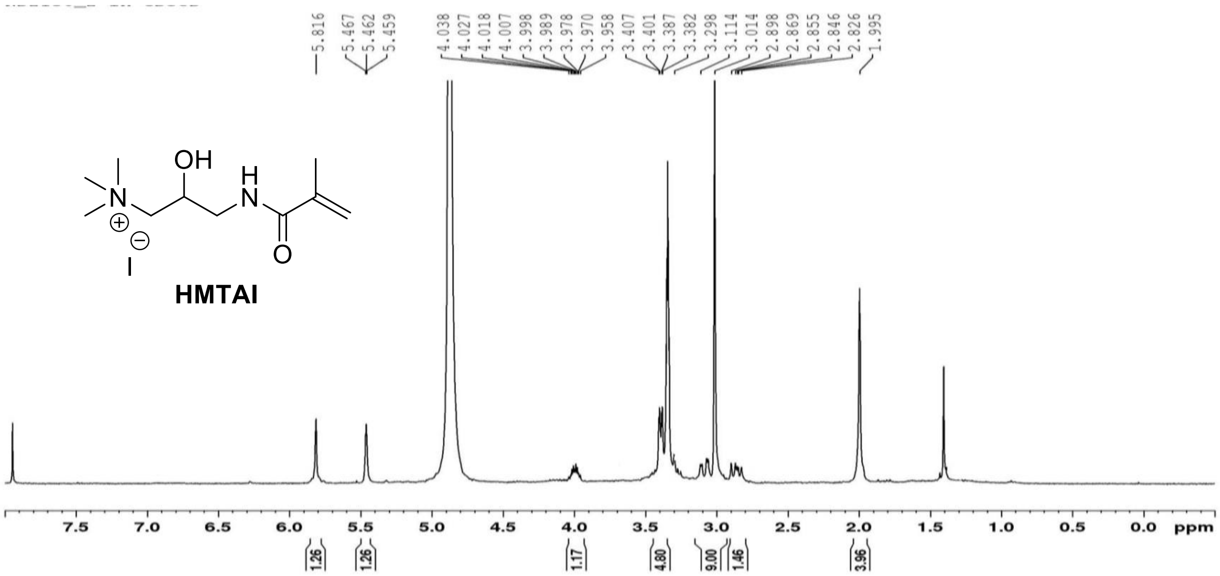

^{13}C (75 MHz) NMR spectrum of **HMTAI** in CD_3OD

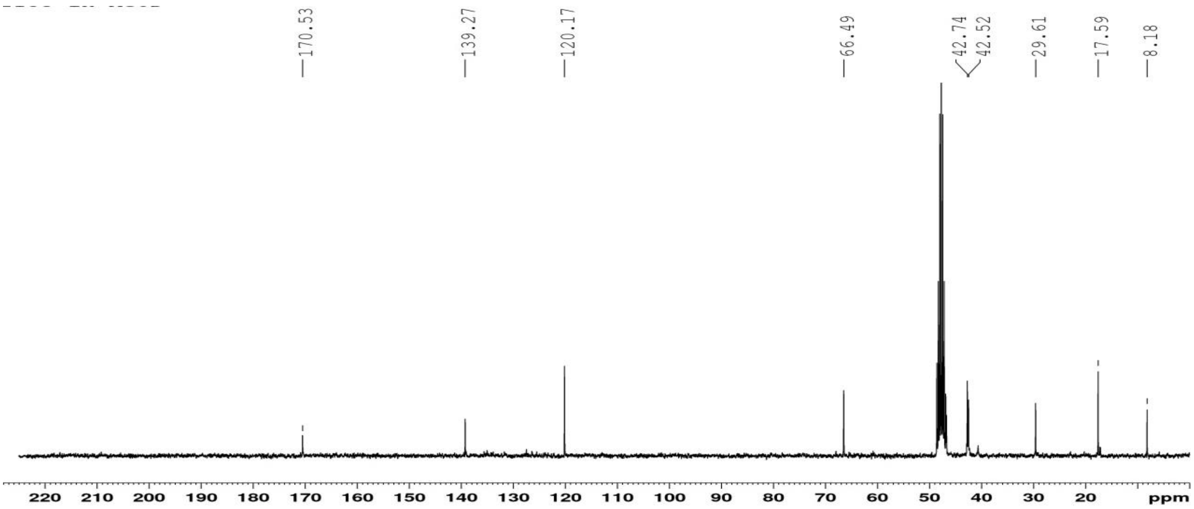

^{1}H (300 MHz) NMR spectrum of **HMTAF** in CD_3OD

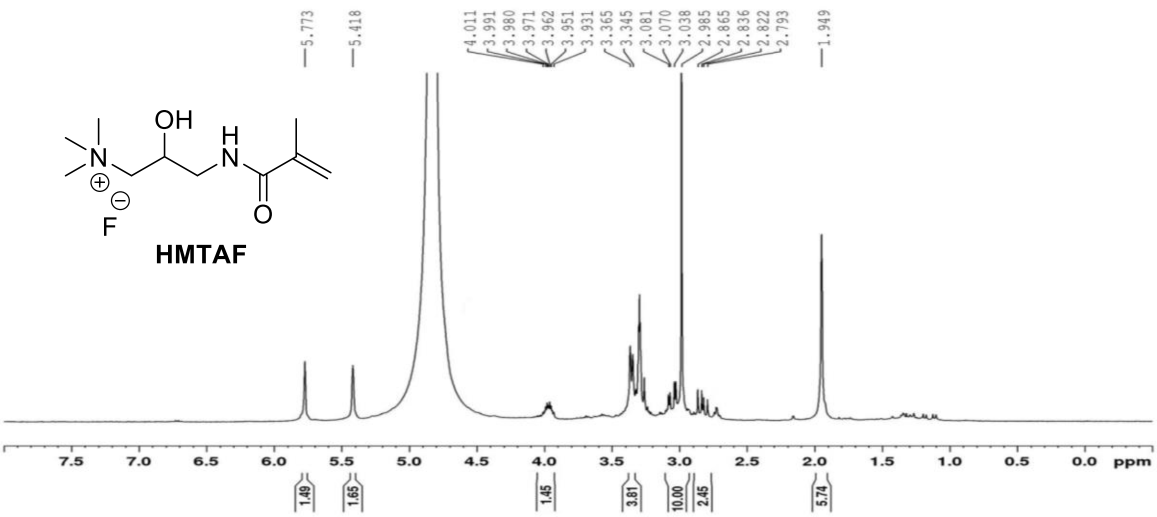

^{13}C (75 MHz) NMR spectra of **HMTAF** in CD_3OD

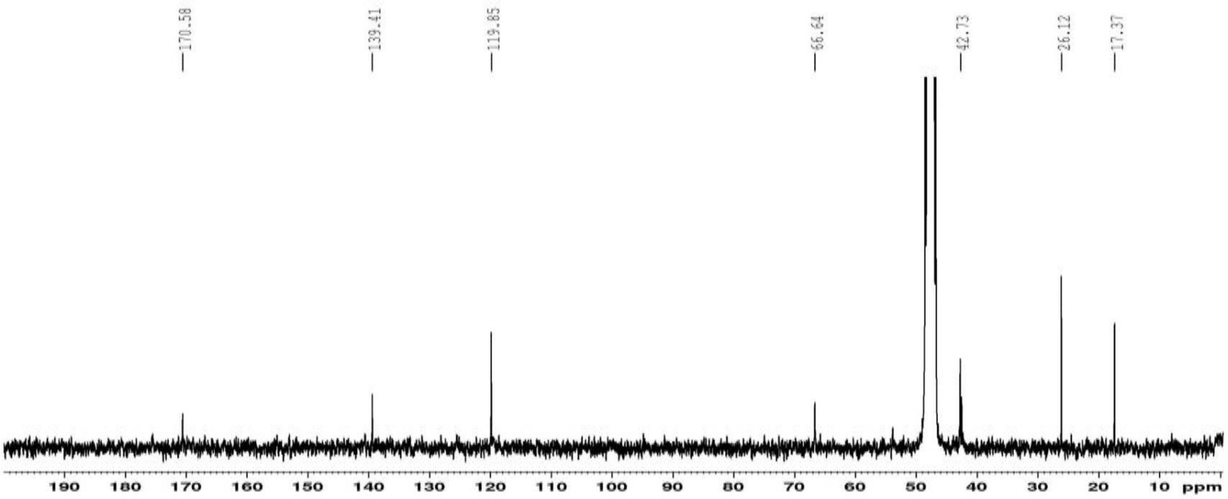

^{1}H (300 MHz) NMR spectra of compound **6** in $CDCl_3$

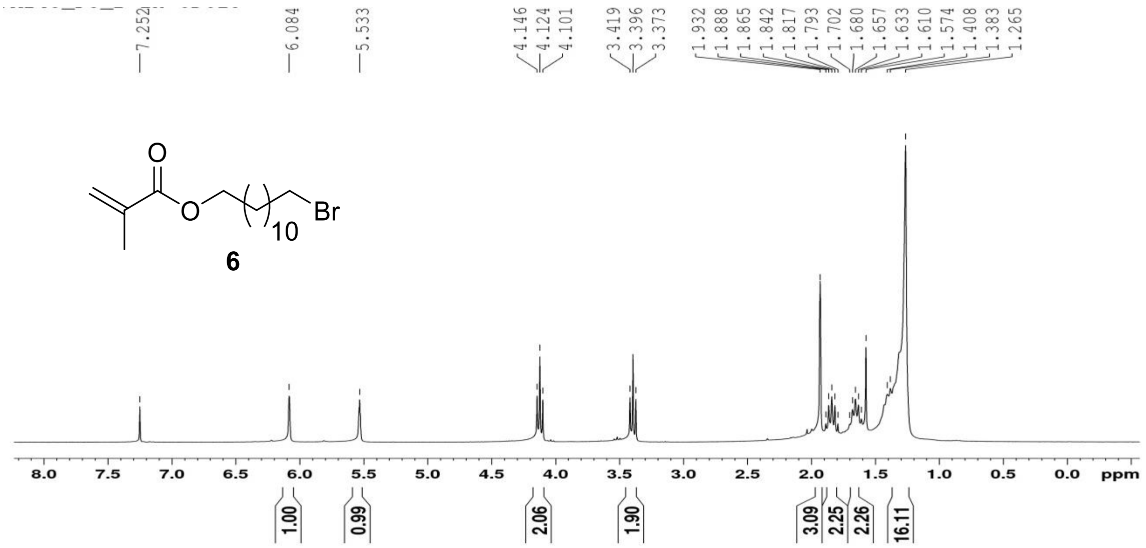

^{13}C (75 MHz) NMR spectra of compound **6** in $CDCl_3$

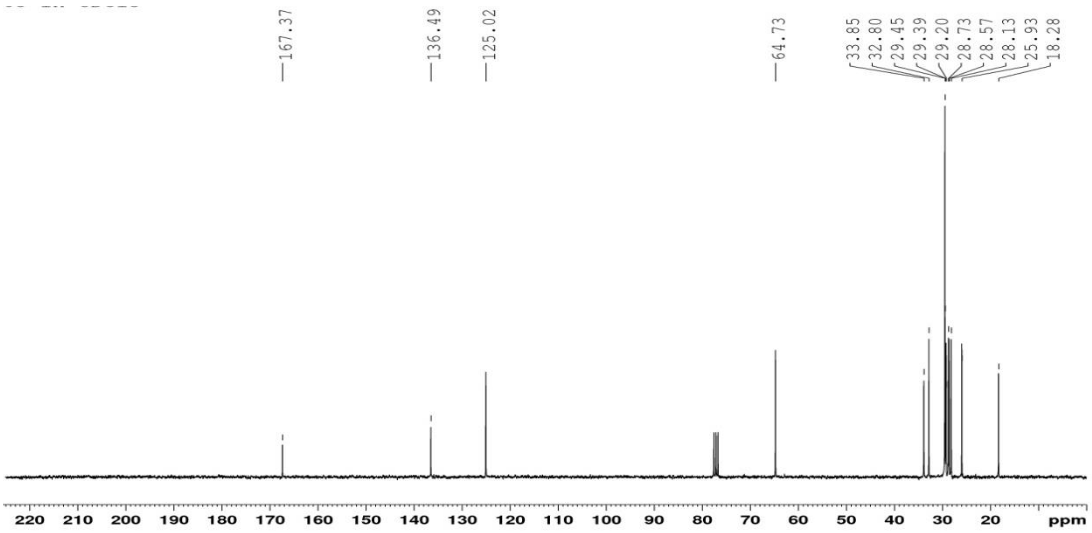

^{1}H (300 MHz) NMR spectra of **MDPB** in $CDCl_3$

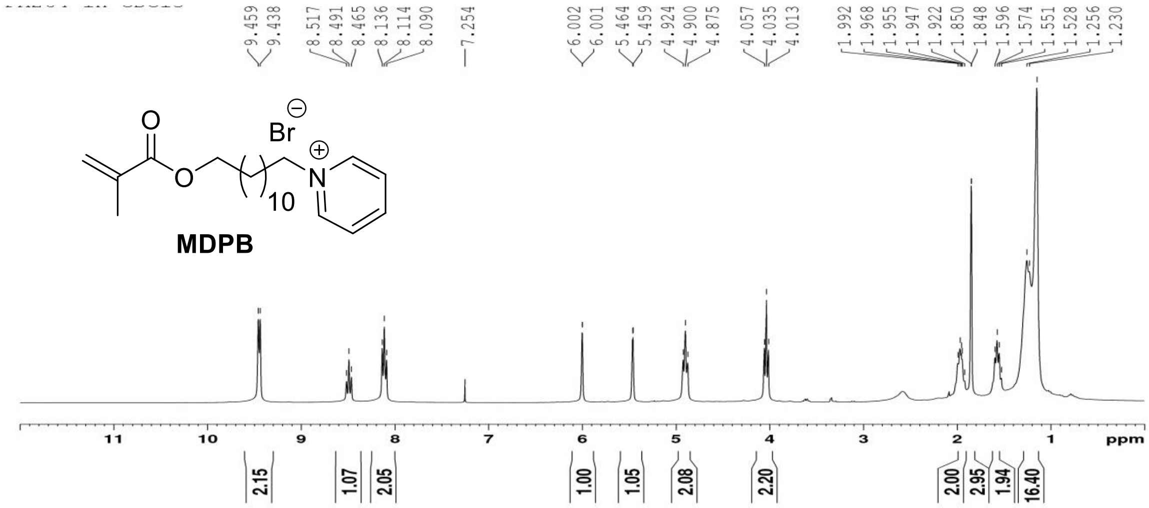

^{13}C (75 MHz) NMR spectra of **MDPB** in $CDCl_3$

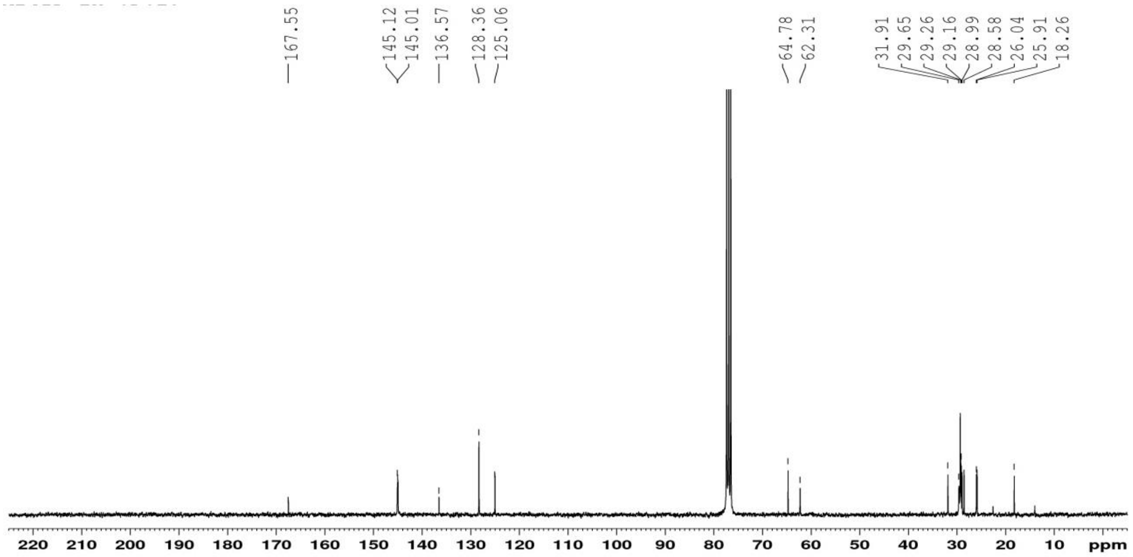

6. Reference

1. Perrault WR, Pearlman BA, Godrej DB, Jeganathan A, Yamagata K, Chen JJ, Lu CV, Herrinton PM, Gadwood RC, Chan L, Lyster MA, Maloney MT, Moeslein JA, Greene ML, Barbachyn MR. The synthesis of *N*-Aryl-5(*S*)-aminomethyl-2-oxazolidinone antibacterials and derivatives in one step from aryl carbamates. *Org. Proc. Res. Dev.* 2003; 7: 533-546.
